# Emerging Classes of Small Non-Coding RNAs With Potential Implications in Diabetes and Associated Metabolic Disorders

**DOI:** 10.3389/fendo.2021.670719

**Published:** 2021-05-10

**Authors:** Cécile Jacovetti, Mustafa Bilal Bayazit, Romano Regazzi

**Affiliations:** ^1^ Department of Fundamental Neurosciences, University of Lausanne, Lausanne, Switzerland; ^2^ Department of Biomedical Sciences, University of Lausanne, Lausanne, Switzerland

**Keywords:** PIWI-interacting RNAs (piRNAs), small nucleolar RNAs, tRNA-derived small RNAs (tRFs), diabetes, metabolism

## Abstract

Most of the sequences in the human genome do not code for proteins but generate thousands of non-coding RNAs (ncRNAs) with regulatory functions. High-throughput sequencing technologies and bioinformatic tools significantly expanded our knowledge about ncRNAs, highlighting their key role in gene regulatory networks, through their capacity to interact with coding and non-coding RNAs, DNAs and proteins. NcRNAs comprise diverse RNA species, including amongst others PIWI-interacting RNAs (piRNAs), involved in transposon silencing, and small nucleolar RNAs (snoRNAs), which participate in the modification of other RNAs such as ribosomal RNAs and transfer RNAs. Recently, a novel class of small ncRNAs generated from the cleavage of tRNAs or pre-tRNAs, called tRNA-derived small RNAs (tRFs) has been identified. tRFs have been suggested to regulate protein translation, RNA silencing and cell survival. While for other ncRNAs an implication in several pathologies is now well established, the potential involvement of piRNAs, snoRNAs and tRFs in human diseases, including diabetes, is only beginning to emerge. In this review, we summarize fundamental aspects of piRNAs, snoRNAs and tRFs biology. We discuss their biogenesis while emphasizing on novel sequencing technologies that allow ncRNA discovery and annotation. Moreover, we give an overview of genomic approaches to decrypt their mechanisms of action and to study their functional relevance. The review will provide a comprehensive landscape of the regulatory roles of these three types of ncRNAs in metabolic disorders by reporting their differential expression in endocrine pancreatic tissue as well as their contribution to diabetes incidence and diabetes-underlying conditions such as inflammation. Based on these discoveries we discuss the potential use of piRNAs, snoRNAs and tRFs as promising therapeutic targets in metabolic disorders.

## Introduction

The publication in 2001 of the entire Human Genome Sequence (1, 2) has provided new insights into the biological relevance of our genes and of their transcripts. Beside the genes that code for proteins, which represent less than 2% of the genome, more than 90% of the other sequences, considered until recently “junk” DNA, were found to be transcribed and to constitute a commensal source of RNAs of all types (3). RNAs can be classified according to their biological function and their physico-chemical properties (protein-coding messenger RNAs, ribosomal RNAs or transfer RNAs) but also according to their size (4). They are classified as small non-coding RNAs if they are shorter or long non-coding RNAs (lncRNAs) if they are longer than 200 nucleotides. Small non-coding RNAs include microRNAs (miRNAs), transfer-derived RNAs (tRFs), small nucleolar RNAs (snorRNAs), small nuclear RNAs (snRNAs), small interfering (siRNAs) and PIWI-interracting RNAs (piRNAs) while lncRNAs include also circular RNAs (cirRNAs). Some non-coding RNAs, such as miRNAs, have been extensively studied during the last two decades and their contribution to the development of a variety of pathological conditions is now well established (5, 6). For others, such as tRFs, snoRNAs, snRNAs, piRNAs, and cirRNAs, a key role in the regulation of numerous transcriptomic, epigenetic and proteomic events is becoming increasingly evident and an involvement in chronic human diseases, including in the etiology of diabetes and its complications, is now emerging (7).

Diabetes is a metabolic disease that currently affects 1 in 11 people worldwide, or 463 million adults, and its incidence is rising, in relationship with unhealthy lifestyles in association to genetic predisposition, and is foreseen to reach 700 million by 2045 (https://www.idf.org/). Having diabetes, strongly impacts the life quality and expectancy of patients due to increased risk of cardiac, vascular, renal and neuropathic complications. Diabetes is characterized by chronic hyperglycemia resulting from the body’s inability to meet its insulin requirements. In type 1 diabetes (T1D), which accounts for approximately 10% of diabetes cases, an autoimmune response directed against pancreatic β-cells leads to a drastic loss of insulin-secreting cells and a near complete lack of the hormone (8). In type 2 diabetes (T2D), representing approximately 90% of the diabetes cases, patients present a loss of β-cell function and/or a resistance of the target tissues (liver, muscle and adipose tissue) to insulin action (9). In order to maintain stable and homeostatic glycemic levels (below 1.4 g/l 1.5 hours after a meal), T1D patients require subcutaneous injections of insulin while individuals suffering from T2D take oral medications to improve insulin sensitivity, in some cases supplemented with insulin injections. Despite major advances in islet transplantation, the manufacture of β-cell surrogates from stem cells, and insulin molecules with an action very close to that of endogenous human insulin, there is at present no curative treatment for this pathology. In this field, research is very active with the hope to better characterize all the mediators and actors involved in the control of β-cell function in order to develop therapeutic strategies that target specifically the dysfunctional pathways responsible for the disease.

Herein, we will focus on three classes of small non-coding RNAs, piRNAs, snoRNAs and tRFs that have so far been less intensively studied, especially in the context of pathologies associated with metabolic disorders. Nevertheless, in view of the available information about their biological relevance, their mechanism of action and their regulation, there is growing interest for the involvement of these three classes of ncRNAs in human diseases, including in metabolic disorders.

In this review, we will discuss the biogenesis of piRNAs, snoRNAs and tRFs while emphasizing on novel sequencing technologies that allow ncRNA discovery and annotation. We will also present the mechanisms of action described so far for each of these categories of small non-coding RNAs. Finally, we will focus on current studies that demonstrate a role for piRNAs, snoRNAs and tRFs in the pathogenesis of diabetes and associated metabolic disorders.

## Biogenesis and Profiling of piRNAs, snoRNAs and tRFs

### piRNA Biogenesis

P-element Induced Wimpy Testis (PIWI)-interacting RNAs (piRNAs) are 21-35 nucleotide-long RNAs that interact with the Piwi subfamily of Argonaute proteins. piRNAs are typically phosphorylated at 5’ end, and 2-O-methylated at the 3’ end. The piRNA pathway is highly enriched in foetal germ cells where it is required for repressing transposon activity, thereby maintaining genome integrity and ensuring faithful gametogenesis (10). A second cluster of piRNAs is expressed in postnatal sperm cells and lack transposon sequences. These piRNAs are reported to function like miRNAs and regulate meiotic gene expression (11). Accordingly, a growing body of evidence suggests somatic expression and function of piRNAs (12–14).

Unlike other Argonaute-associated small ncRNAs, such as miRNAs and short interfering RNAs (siRNAs), piRNAs originate from single-stranded RNA precursors called piRNA clusters and do not require the endoribonuclease Dicer for maturation. In most metazoans, piRNA loci are unidirectionally transcribed (15). These piRNA precursors are transported from the nucleus to the cytoplasm where they are matured into primary piRNAs through a series of enzymatic processes. First, the secondary structures of the piRNA precursors are resolved by the RNA helicase Moloney leukemia virus 10-like 1 (MOV10L1). Subsequently, the unwound piRNAs are cleaved by the endonuclease Zucchini (Zuc), generating pre-piRNAs containing a 5’ monophosphate. These pre-piRNAs are loaded onto PIWIs that facilitate the exonuclease-mediated trimming, producing piRNAs of specific lengths. Finally, Hen1, an RNA 2-O-methyltransferase, methylates the 2’ oxygen at the 3’ end of the piRNA, which stabilizes the piRNA structure (15). In the germline, these primary piRNAs are amplified further by a biogenesis cycle termed ping-pong loop (16). This cytoplasmic series of events are initiated when these primary piRNAs bind to the Aub subclass of Argonaute proteins. As Aub-piRNA complex induces endonucleolytic cleavage of the target transposons, they also generate sense-strand secondary piRNAs. Secondary piRNAs are incorporated by Ago3 and used for cleaving piRNA cluster transcripts, producing precursor piRNAs that are fed into the loop (16).

### piRNA Detection

Identification and annotation of new piRNAs usually rely on immunoprecipitation of PIWI and analysis of pulled down molecules by RNA-sequencing (17, 18). However, this method is laborious and can be insensitive for detecting lowly expressed piRNAs. Computational methods provide a viable option for *de novo* piRNA annotation. The initial algorithm built to predict novel piRNAs was based on *k-mer* motifs (19). This algorithm provides >95% accuracy and >60% sensitivity. Subsequently, Piano and piRNAPredictor, software based on support vector machine and weighted ensemble, were developed (20, 21). Although they are 95% accurate in predicting transposon-related piRNAs, they are not optimized to detect somatic piRNAs. Alternatively, piRPred uses a combination of sequence features (*k-mer* motifs and 5’ uridine position) and genomic information to achieve 86% and 89% prediction accuracy in human and fruit fly data, respectively (22). Recently, piRNN, a program based on a convolutional neural network classifier and *k-mer* encoding strategy was developed using *C. elegans*, *D. melanogaster*, rat and human piRNAs as training models. This tool is reported to achieve at least 94% accuracy, precision, and specificity, highlighting the advancement of bioinformatic tools in piRNA annotation (23).

### snoRNA Processing Pathways

SnoRNAs are intermediate-sized (60-300nt), closed-loop, non-polyadenylated RNA molecules, well-conserved, mainly located in the nucleolus and detected in all eukaryotic organisms (24, 25). The nucleolus is the largest and most studied nuclear body, high place of ribosomal RNA (rRNAs) synthesis and processing and ribosome assembly (26, 27). SnoRNAs play a vital role by ensuring modifications such as pseudouridylation (Y) (class 1 of snoRNAs) or 2’-O-methylation (2’Ome) (class 2 of snoRNAs) on different RNA types, mainly rRNAs, tRNAs and small nuclear RNAs (snRNAs). These two classes of RNAs account for more than 85% of cellular RNA and their modifications are needed for their stability and function, thus giving snoRNAs a primordial place (28).

In yeast and plants snoRNAs are mostly transcribed from their own independent genomic regions, whereas in animals most snoRNA-coding sequences are intronic. When they are generated from independent DNA regions, snoRNAs are transcribed from monocistronic (under control of their own promoters *via* RNA polymerase II or III) or polycistronic (via RNA polymerase II) regions (25, 29). The 5’ end of the transcripts is either removed or given a trimethylguanosine (m2,2,7G) cap before undergoing a maturation process of the 3’ end ensuring the production of mature and functional snoRNAs. RNA polymerase II ensures the transcription of snoRNAs produced from introns under the control of the promoters of their host genes. In this case, snoRNA transcripts undergo splicing and debranching of the snoRNA-containing intron followed by trimming by exonuclease enzymes (29, 30).

SnoRNAs are classified into two categories defined according to highly conserved sequences known as “boxes”, which are present in pairs on each snoRNA molecule (31). This allows to distinguish C/D and H/ACA box snoRNAs. The structure of C/D snoRNAs consists of a large closed loop which includes a C box (RUGAUGA motif) and a D box (CUGA motif) as well as a C’ and a D’ box, which are less conserved. H/ACA snoRNAs consist of two stem loops connected by the H box (ANANNA motif) and an ACA sequence in 3’ position. An additional category of ncRNAs termed Cajal body-associated RNAs (scaRNAs) located in sub-nuclear structures known as Cajal bodies (32) are derived from either C/D snoRNAs displaying a long UG repeat or H/ACA snoRNAs including an additional UAGA pattern (CAB box) (24, 33, 34). The absence of features such as poly(A)tail and m7G cap at the 5’ end, which are structural hallmarks of mRNAs and facilitate their export into the cytosol, may explain why snoRNAs remain exclusively localized in the nucleus (35, 36).

Irrespective of the class to which they belong, snoRNAs associate to proteins and display a stable ribonucleoproteic structure (37). Due to their compact entanglement with protein snoRNPs, snoRNAs are highly stable structures compared to other transcripts produced by Pol II.

### snoRNA Identification

The number of genes transcribed into snoRNAs increases proportionally with the complexity of the organisms, with less than a hundred genes coding for 76 snoRNAs in *Saccharomyces cerevisiae* to more than 500 snoRNA genes in humans (38, 39). The techniques used to detect snoRNAs are critical to ensure the specificity of the identification (40). SnoRNAs are easily quantifiable by conventional qPCR after being reverse transcribed (RT) by random priming. Although, RNA-sequencing in combination with bioinformatics allow a very accurate estimation of gene expression and the *de novo* construction of the transcriptomic landscape, conventional approaches such as semi-quantitative northern blotting and RNase protection assay (RPA) are still being used to validate genomic analyses (41). *In silico* techniques estimate the population of snoRNAs to exceed thousands of candidates in mammalian cells (42, 43). The majority of human snoRNAs are produced from mRNA introns or lncRNAs. Several studies have observed that the expression of snoRNAs released from spliced introns does not always correlate with the mRNA level of the host gene (44–46). A large proportion of the host genes from which snoRNAs are transcribed code for mRNAs or lncRNAs that are non-functional and are destined to be degraded by the nonsense-mediated RNA decay pathway (44). This makes snoRNA production an actively regulated process for functional and biological purposes. This area of research still includes many open questions and further studies will be necessary to clarify the relationship between snoRNAs and their hosting genes.

### tRF Synthesis

Transfer RNAs (tRNAs) are 73-90 nucleotide long RNAs that help decode mRNA sequences into proteins. Their role as amino acid carriers during protein translation is well documented. tRNAs are the most abundant cellular RNAs, comprising approximately 12% of total RNA in most cells (47, 48). Due to the degeneration of the genetic code, several codons code for the same amino acid. Indeed, the standard genetic code consists of 64 codons corresponding to 20 different amino acids. There are more than 260 different tRNAs in humans that are generated from more than 400 nuclear genes (49). Furthermore, mitochondrial DNA encodes for 22 additional tRNAs (mt-tRNAs) (50). This large number of tRNA molecules is due to the presence of isoacceptors (tRNAs carrying the same amino acid but consisting of a different nucleotide sequences in the body and in the anticodon region) and isodecoders (tRNAs sharing the same anticodon and carrying the same amino acid but with a different nucleotide sequence in the body). Isoacceptors and isodecoders are present in several copies in the genome and their expression (constituting the tRNA pool) is tissue and cell type-specific and can be modulated by stress conditions. Post-translational tRNA modifications introduce further complexity to tRNA function. These modifications may occur in the nucleus, cytoplasm and mitochondria through the action of various tRNA modifying enzymes, and modulate base pairing, tRNA folding and/or stability (51). The average mammalian cytoplasmic and mitochondrial tRNAs contain 13 and five modified bases, respectively. tRNAs are the most stable RNAs *in vivo*, but hypomodified tRNAs are targeted for degradation (52). In addition to the degradation of dysfunctional tRNAs, tRNAs may also undergo endonuclease-mediated fragmentation.

**Figure 1:**
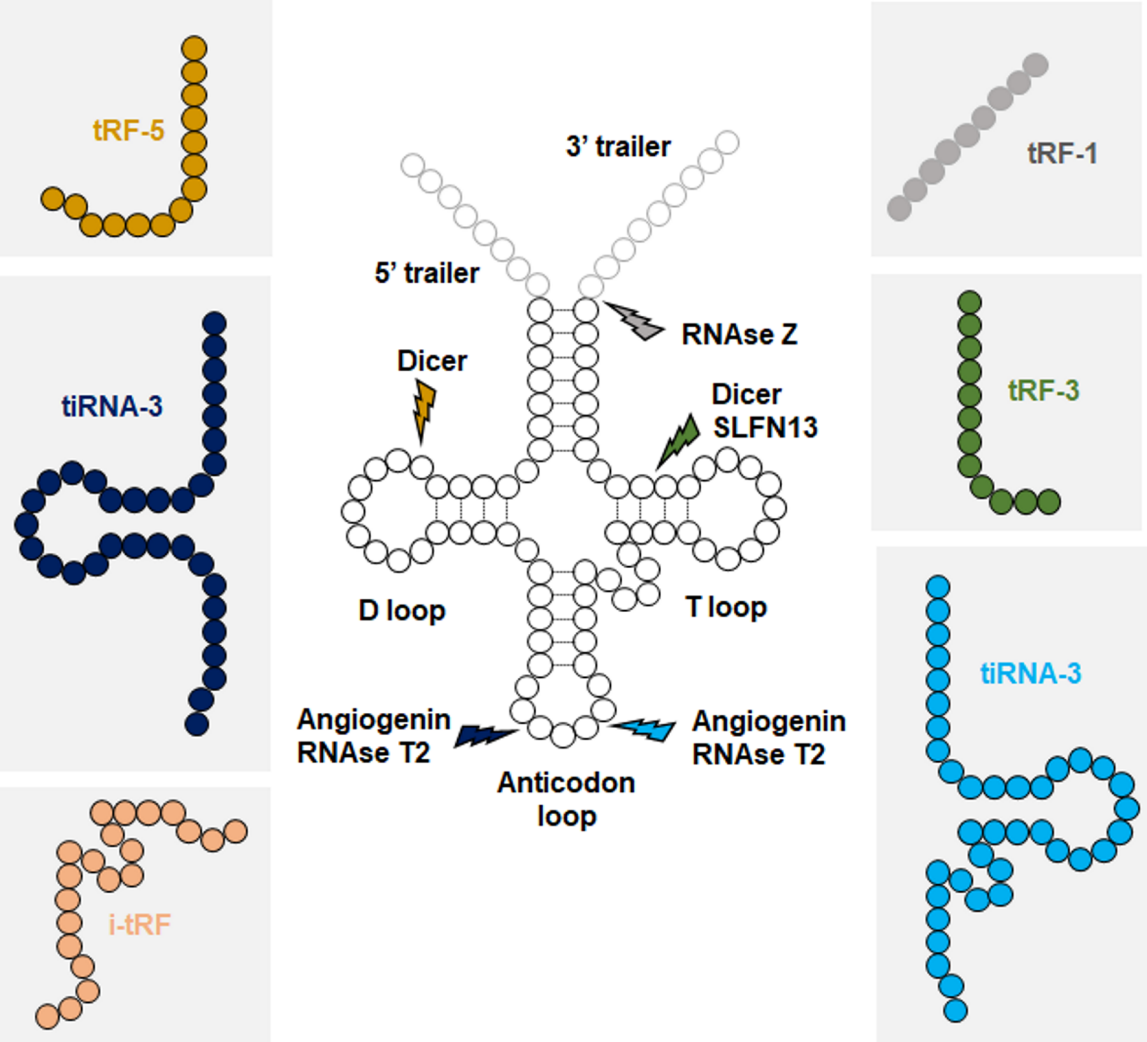
Biogenesis of tRFs. Each class of tRFs are categorized based on where they map on tRNA sequence. tRF-1s are generated from trailer sequences present on primary tRNA transcripts (pre-tRNAs), while other classes are generated from mature tRNAs. Approximate cleavage positions and the responsible endonucleases for each class of tRFs are indicated.

Both pre-tRNAs and mature tRNAs can be fragmented in concert with tRNA expression, or in response to a stress stimulus (53–56). While the nomenclature of tRFs is not yet well established, six main classes of tRFs have been described. These classifications are based on the tRNA fragmentation site: tRF-1s are generated from pre-tRNAs, tRF-3/5s from distal ends of mature tRNAs, tiRNA-3/5s from the cleavage of anticodon sites of mature tRNAs, and i-tRFs from internal sequences of mature tRNAs (**Figure 1**). While Angiogenin is the main enzyme responsible for cleaving the anticodon site, different endonucleases have been reported to generate other classes of tRFs, including Dicer, SLNF13, RNase T2, and RNase Z (30, 55, 57).

### Detection and Annotation of tRFs

Like other ncRNAs, detection of tRFs has been substantially improved by the advancement of high-throughput small RNA sequencing. The fragments can be annotated by aligning the sequencing data to mature and pre-tRNA sequences using publicly available genomic tRNA databases, such a GtRNAdb (58). This permitted to reveal the tRF landscape under various physiological and pathological conditions, paving the way for functional studies (59). However, detection of tRFs by small-RNA sequencing has certain limitations that need to be overcome. First, certain post-translational modifications that decorate tRNAs, such as 3’ end aminoacylation and various methylations, may interfere with adaptor ligation and cDNA synthesis, leading to sequencing biases (60). To overcome this issue, RNA samples can be pre-treated with specific enzymes that remove these post-translational modifications (60, 61). Second, tRFs detected by sequencing need to be distinguished from random degradation products of tRNAs. Genuine tRFs can be predicted by using a binomial test, a statistical method that normalizes the tRF levels with theoretically expected levels of degradation products (62). Finally, the presence of tRNA isodecoders which share extended regions of similarity can prevent the identification of the parent tRNA (63).

Concurrent with the growing high-throughput data, several tRF databases and bioinformatic tools have been developed. Databases such as MINTmap and tRF2Cancer extract and catalogue raw and normalized tRF levels from published human small RNA-seq data under physiological and pathological conditions (62, 63). tRF2Cancer and sRNAtools further provide tRF identification from user uploaded sequencing data (64).

## Biological Roles of piRNAs, snoRNAs and tRFs and Methods to Determine Their Functions

### Mechanisms of piRNA Function

The ancestral function of piRNAs in most animals is to provide post-transcriptional control of mRNA transposons and thus repress transposon mobilization in order to perpetuate and protect the genome of germline cells (65). This mode of repression relies on the production of piRNAs *via* the aforementioned “ping-pong” process. Guided by the piRNAs, PIWI proteins cleave the targeted mRNAs 10 nucleotides upstream of the 5’ end of the piRNA as a guide, leading to the formation of a new piRNA in the opposite direction from the 5’ end. Thus, piRNAs belonging to the same pair generated by the ping-pong ring share 10 nucleotides at their 5’ end (10). Nevertheless, many piRNAs have been identified that do not present any complementarity to transposable elements (TE), suggesting that they may have a different mechanism of action (66).

The presence of transposons within the genome represents a potential risk of generating illegitimate recombinations, the appearance of double-stranded DNA breaks following transposon replication, their aleatory insertion causing an alteration in the sequence of coding genes as well as the ectopic expression of neighboring genes under the control of the promoter of the inserted transposons (67, 68). piRNAs exerts a protective effect against the insertion of transposons mainly in germ cells. They act by binding to PIWI proteins and guiding them to the transcribed nascent transposon, which are then silenced (65). A large proportion of piRNAs are thus generated from TE sequences and target TE transcripts. However, a subset of piRNAs does not emanate from TE sequences but are also produced during spermatogenesis. These piRNAs, called pre-pachytene, are present in male gonad germ cells before birth and during the neonatal period and silence the TE using the mechanism described above (67). Conversely, non-TE piRNAs, which appear after birth, have a different mode of action and direct PIWI proteins to cleave and affect the stability of targeted mRNAs (67). However, the function of the piRNAs that do not target TE remains unclear (69).

**Figure 2:**
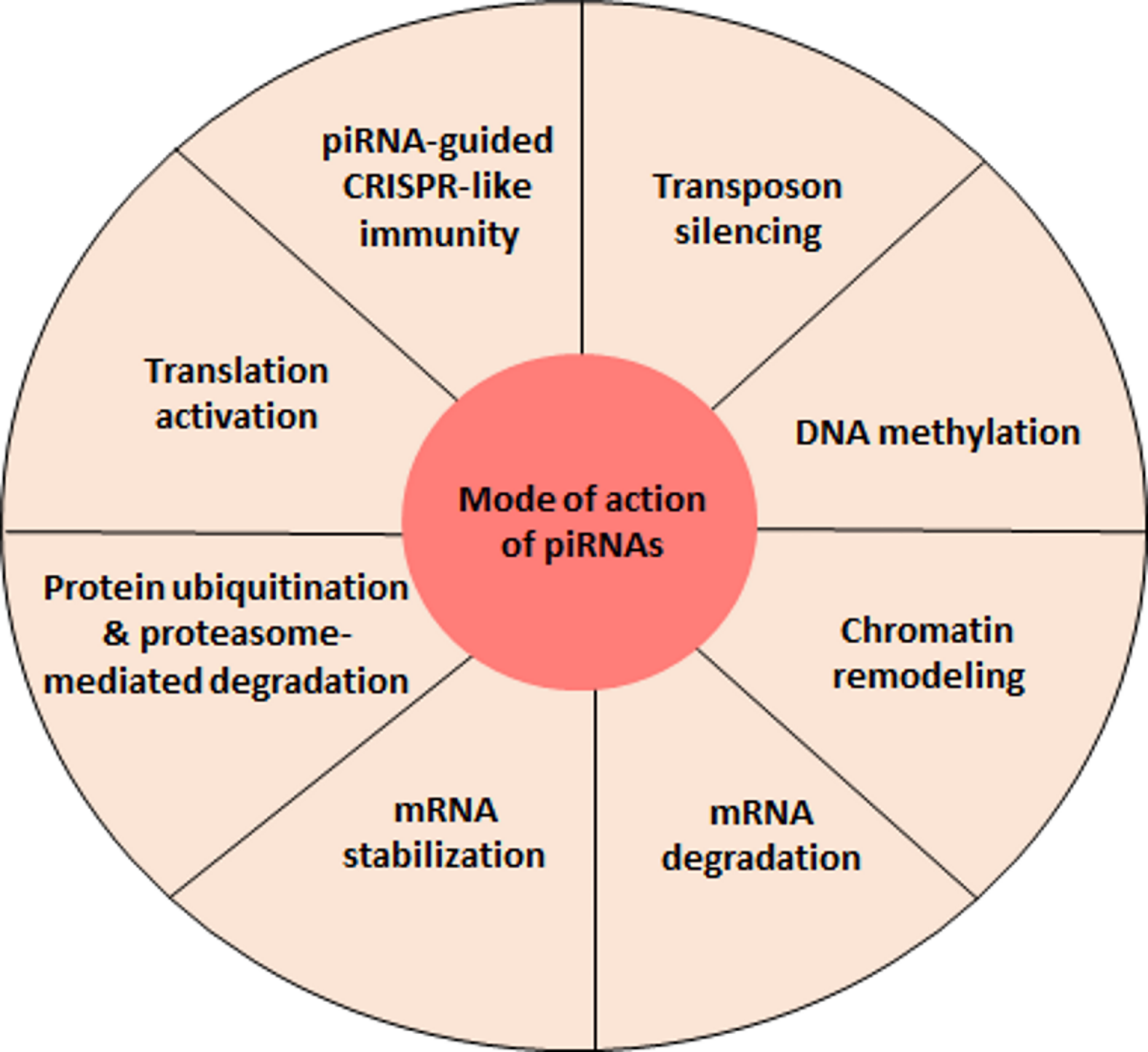
piRNA-mediated regulatory functions. At present, the best characterized function of piRNAs is the repression of transposable elements. However, recent evidence indicates that piRNAs can accomplish a variety of other regulatory activities, findings that will most certainly be further strengthen by future studies.

Nevertheless, piRNAs are also produced in somatic cells where the preservation of their ancestral function seems to be questionable. For example, piRNAs expressed in invertebrate somatic cells seem to play an essential role in fighting against viral infections (65, 70, 71). The mechanism at stake here follows the integration of the viral genome which would trigger the ping-pong cycle and thus induce the production of piRNAs that recognize viral RNA causing to its degradation (71). This model is mainly described in mosquitoes and requires further research to confirm its validity in other organisms. The studies performed during the last 20 years focused on the understanding of piRNA biogenesis but also on their mode of action and their role within germ and somatic cells. However, many questions remain open. In particular, the emergence of piRNA functions that are independent of their capacity to repress transposons will need to be further explored (**Figure 2**).

### Strategies to Unveil piRNA Targets

The function of piRNAs which do not act on transposons remain largely unknown. The limited knowledge in the field also hinders the development of prediction tools for piRNA targets. Exceptionally, piRNA targeting dynamics has started to be unveiled in *C. elegans* (72). Additionally, transcriptome-wide interactions between piRNAs and their targets have been identified using an *in vivo* cross-linking approach (CLASH) (73). Thus, these studies enabled the construction of pirScan and piRTarBase, target prediction tools for piRNAs in *C. elegans* (74, 75). With the rapid evolution in the piRNA field, similar tools are expected to be built for mammalian piRNAs, potentially contributing to unravel the function of piRNAs under normal and pathophysiological β-cell conditions.

### snoRNAs: Functional Relevance

**Figure 3:**
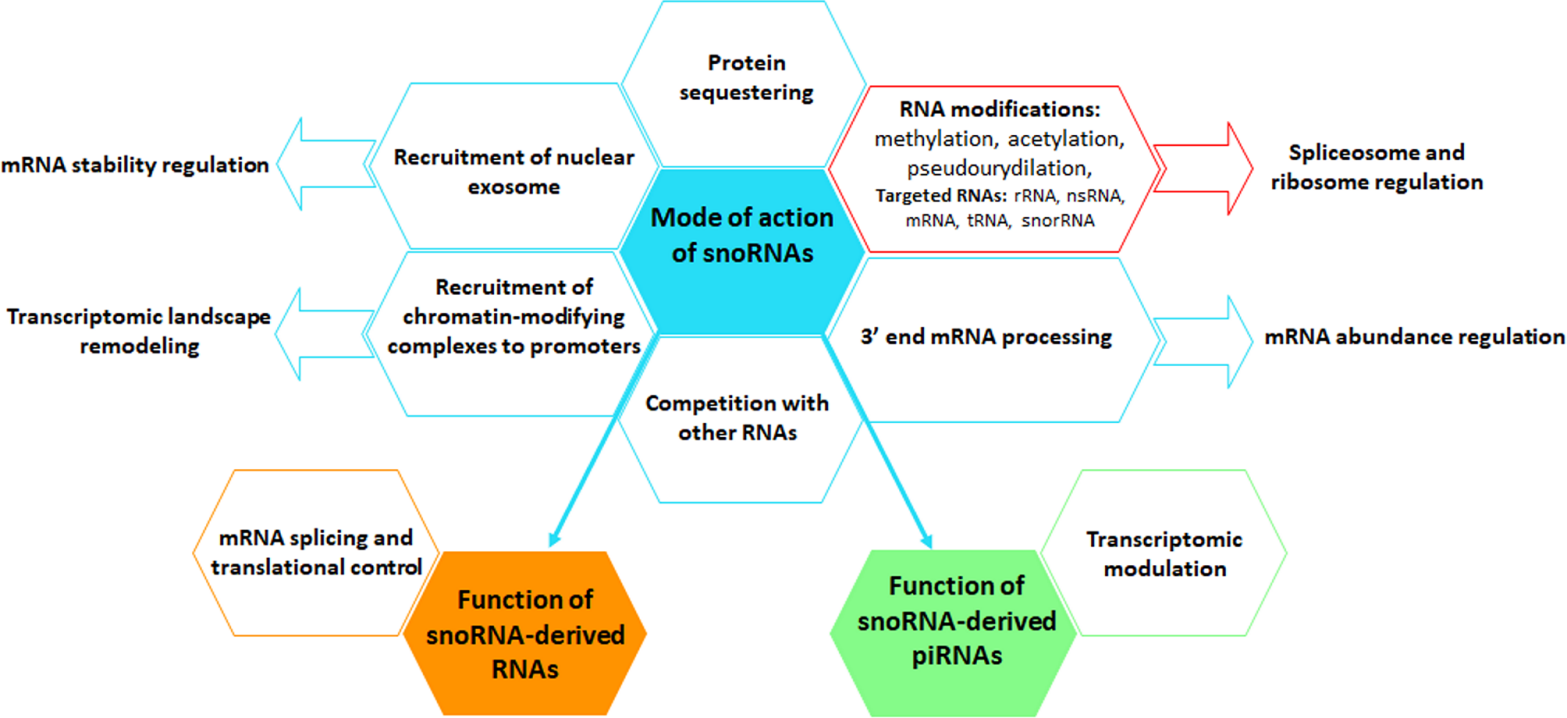
Graphical representation of the mode of action of snoRNAs. snoRNAs perform multiple canonical (red box) and non-canonical (blue boxes) functions, using a broad spectrum of mechanisms, including the regulation of the level and activity of mRNAs and other types of RNAs, protein biogenesis and protein availability. snoRNAs can also be cleaved into snoRNA-derived RNAs, named sdRNAs, that interfere with mRNA splicing and translational processes or into piRNAs, thus regulating the transcriptome. Arrows indicate biological functions that are controlled by snoRNAs.

The canonical function of both snoRNAs and scaRNAs is to implement modifications, mainly methylations and pseudouridylations. SnoRNAs preferentially target ribosomal RNAs (rRNAs) and small nuclear RNAs (snRNAs), in order to ensure their maturation in the nucleus, protect them from nucleolytic degradation, thus indirectly affecting the function of the ribosome and of the spliceosome (25). The vast majority of the snoRNAs carry specific sequences complementary to various types of cellular RNAs (short sequences of 7 to 21 nucleotides called antisense element (ASE) or guide region) ensuring recognition and fine-tuning of substrate RNAs (76). SnoRNAs associate with proteins forming small nucleolar ribonucleoprotein (snoRNP) complexes. SnoRNA partner proteins are essential for their biogenesis and to ensure their stability by protecting them from exonuclease, but also to maintain their nuclear localization (76). C/D snoRNAs induce 2-O’-methylation of targeted rRNAs at specific sites. C/D snoRNAs form snoRNPs complexes mainly with Snu13, Nop56, Nop58 and fibrillarin methyltransferase proteins. H/ACA snoRNAs induce pseudo-uridyl modifications by catalyzing the isomerization of uridine to pseudouridine. The protein partners of H/ACA boxes are the dyskerin, Nhp2, Nop10 and Gar1 factors. SnoRNAs can also be directly involved in the synthesis of rRNA precursors in the nucleolus (77). The interference of snoRNAs with ribosomes or the biogenesis of snRNAs itself can result in the alteration of protein-coding gene expression *via* alternative splicing or translation control (78). Along this line, a study demonstrated circadian periodicity of snoRNA expression, which is presumed to be at the origin of the rhythmic expression of translation initiation factors, ribosomal proteins and ribosomal RNAs and thus of ribosome biogenesis in mouse liver (79). The capacity of snoRNAs to be cleaved into piRNAs can also indirectly affect the regulation of the genomic and transcriptomic profile (80). A significant number of studies revealed that snoRNAs can exert a broader action *via* new emerging mechanisms including (**Figure 3**): (i) acetylation of rRNAs, (ii) methylation of tRNAs, (iii) competition on binding sites with other types of RNA, (iv) chromatin remodeling, (v) recruitment of the nuclear exosome, affecting the stability of pre-mRNAs, but also (vi) regulation of mRNA abundance via, among other things, the control of mRNA 3′ processing (81). So far, the studies that have established the function of snoRNAs have focused on the dominant role played by snoRNAs in the nucleus; however, the detection of snoRNAs in other cellular compartments suggests that these small ncRNAs may have novel and less conventional functions (82–84).

The function of a non-negligible fraction of the so-called orphan snoRNAs remains unknown due to the lack of complementarity of their ASE with any RNA sequence or to a structure that differs from conventional snoRNAs (shorter or longer or with boxes without consensus motifs or with both C/D and H/ACA boxes) that potentially indicate a different mechanism of action (77, 85, 86). Although certain functions have been attributed to these non-canonical factors (24), their precise role, the type of modifications they may induce and, above all, whether they are expressed at significant levels remains to be explored.

A significant degree of uncertainty remains also about a brand-new class of small RNA molecules that seem to derive from mature snoRNAs (87). Deep sequencing techniques revealed that snoRNAs can be cleaved into smaller and more stable molecules named snoRNA-derived RNAs (sdRNAs) (88–91). The function of sdRNAs remains to be established, although pioneering work suggests an involvement in mRNA splicing and translation control (92, 93). In spite of the huge advances in deep-sequencing, many snoRNAs, sdRNAs or snoRNA-like genes have not yet been annotated and are absent from current databases (40).

### Techniques Used to Decrypt snoRNA Functions

The knowledge acquired so far about the function of snoRNAs has emerged thanks to advanced techniques which we will discuss in the following paragraph. The first approaches used to elucidate the role of snoRNAs were oriented towards the detection and localization of canonical modifications (2’-O-ribose methylation and pseudouridylation) on target RNAs. One strategy consists in performing a reverse transcription (RT) at high concentration of dNTPs (in low concentration of dNTPs, the reverse transcriptase stops at the nucleotide preceding the modification and thus aborts the RT) followed by high-resolution electrophoresis allowing the identification of the modified sites. Another alternative based on a similar principle, consists in using an unbiased high-throughput 2′-O-ribose methylation profiling technique called RimSeq (43). In this case, cDNAs in which the synthesis has been prematurely stopped due to the presence of a limited concentration of dNTP, modifications are sequenced. Finally, another method, called RiboMethSeq, takes advantage of the fact that both pseudouridines previously treated with a CMC compound (carbodiimide) and phosphodiester bonds, located 3′ to a 2′-O-methylated residue, are protected from alkaline hydrolysis. Alkaline hydrolysis induces partial fragmentation of the modified RNAs targeted by the snoRNAs, with preservation of the sites containing the methylations or pseudouridines, thus revealed by sequencing. When modifications in 3’ regions or in several adjacent residues of snoRNAs are present, sequencing reactions are impaired and mass spectrometry techniques were used (94, 95).

Over the last 10 years, there has been an emergence of methods to identify the snoRNA interactome which are very promising. These techniques include CLIP (UV cross-linking and immunoprecipitation) (94, 96, 97), CLASH (cross-linking, ligation, and sequencing of hybrids) (98), PAR-CLIP (photoactivable ribonucleoside-enhanced crosslinking and immunoprecipitation) (99) and hiCLIP (RNA hybrid and individual-nucleotide resolution ultraviolet cross-linking and immunoprecipitation) (100). These methodologies are based on the use of antisense probes ensuring a selective and specific pull-down of the snoRNA of interest. The interactions between the snoRNA molecules are stabilized by exposure to ultraviolet light before proceeding with cell lysis and snoRNA sequencing allowing the identification of the snoRNAs and of their partners. In the technique known as LIGR-seq (ligation of interacting RNA followed by high-throughput sequencing), the cells are treated with the photoreactive psoralen derivative 4′ -aminomethyltrioxsalen (AMT) which captures RNA: RNA interactions engaged in base pairing (101). In PARIS (psoralen analysis of RNA interactions and structures), the use of S1 nuclease/RNase III enzymes induces the fragmentation of AMT-cross-linked RNA which are then analyzed in 2D electrophoresis (102). The SPLASH technique is very similar with the alternative of covalently binding with the RNA by a biotinylated psoralen and capturing Mg^2+^-fragmented cross-linked RNA segments on streptavidin beads before sequencing (103). Finally, one of the latest technical developments has led to the COMRADES method (cross-linking of matched RNAs and deep sequencing) which again consists of a pull-down of cross-linked RNA by the use of a cell-permeable azide-modified psoralen derivative (psoralen-triethylene glycol azide) (104).

Overall, these techniques, which consist in capturing RNA: RNA interactions upstream of sequencing, have been proven effective in the identification of predicted snoRNA targets but also in the discovery of new non-canonical factors targeted by snoRNAs (94, 105, 106).

### Emerging Roles and Mechanisms of Actions of tRFs

**Figure 4:**
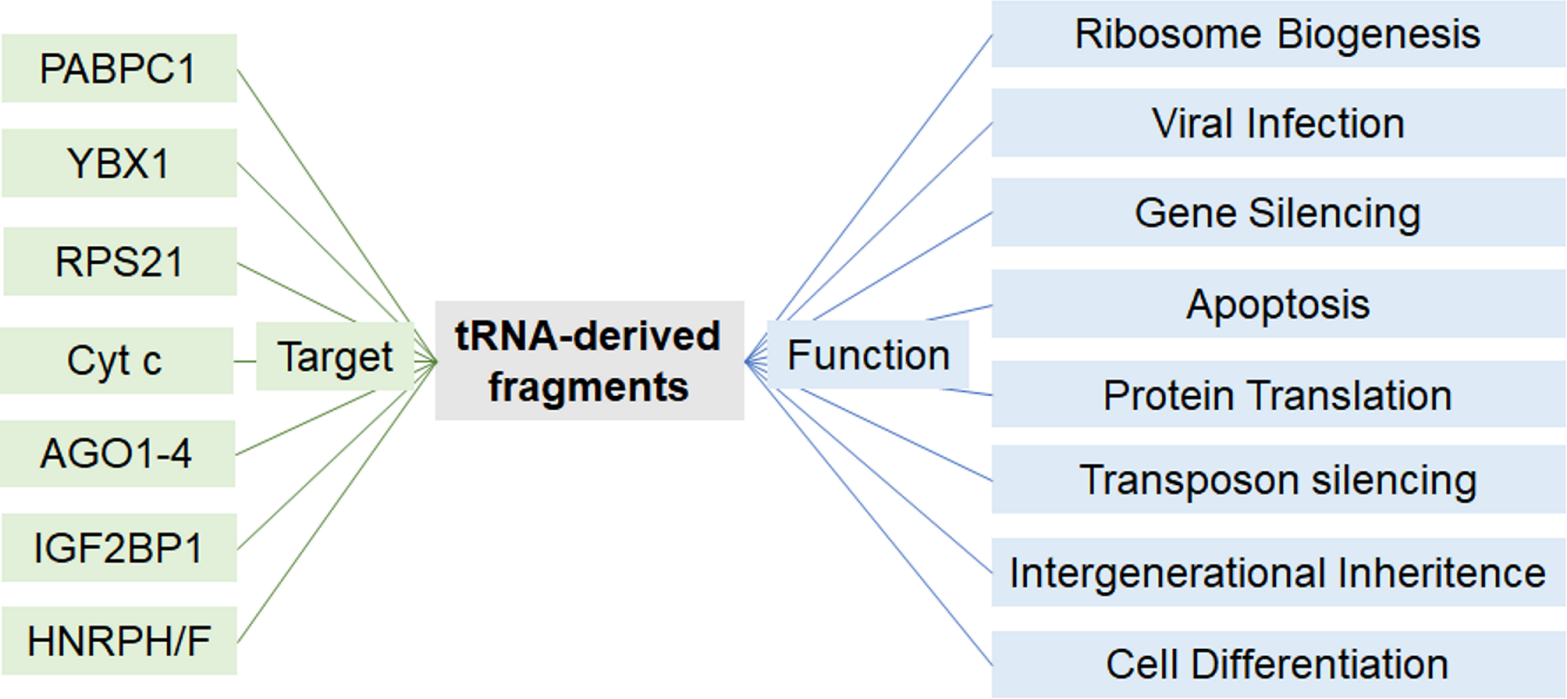
Proposed modes of action of tRFs. Depending on the cellular setting and tRF-type, tRFs may act on different proteins to regulate a variety of cellular functions.

In recent years, concurrent with the advancement of in-depth analyses of deep sequencing data, the implication of tRFs have been described in a wide array of physiological and pathological events including stem cell maturation, organ cross talk, intergenerational inheritance, metastasis, and response to viral infection (**Figure 4**) (59, 107–112). Diverse mechanisms of actions have been described which depend on the tRF type. Dichotomous functions of a single tRF have also been reported in different settings (53, 113). tRFs were shown to associate with proteins of the Argonaute (AGO) family to regulate gene expression, analogous to miRNAs. These interactions were validated in various models *in vivo* and *in vitro*, using photoactivatable ribonucleoside-enhanced crosslinking and immunoprecipitation (PAR-CLIP) of AGO1-4, co-immunoprecipitation (co-IP) of tRFs, and co-IP of various members of the AGO family (114–116).

tRFs are also implicated in translational regulation. In U2OS cells, alanine tiRNA-5 represses translation by interacting with the RNA binding protein YBX1 and displacing eIF4G/A from capped and uncapped mRNA (117). In line with this, YBX1 PAR-CLIP in primary human breast cancer cells revealed binding to a conserved motif within glutamate, glycine, tyrosine, and aspartate i-tRFs, which destabilizes cancer transcripts by displacing YBX1 (109). Importantly, these results were supported by *in vivo* colonization assays in mice, where fine-tuning of tRF levels in xenografts, either by transfection of mimics or antisense LNA inhibitors, impacted metastatic colonization (109).

In sharp contrast to the role of certain tRFs in translational repression, glycine and glutamine tiRNA-5s promote translation by interacting with several ribosomal proteins including the 40S ribosomal protein S21 (RPS21) (118). In this case, RPS21 interaction was first identified by an unbiased proteomic analysis of tiRNA-5 co-IP in HeLa cells, and the interaction was further confirmed when the co-IP was inversely performed, and RPS21 was used as the bait (118). Interestingly, the role of tRFs in ribosomal function was extended further when a leucine tRF-3 was shown to bind to RPS28 and RPS15 mRNAs to enhance their translation, an essential process for hepatocellular carcinoma development as illustrated both *in vitro* and in patient-derived orthotopic hepatocellular carcinoma model in mice (110).

### Databases and tRF Target Predictions

Computational predictions of tRF targets have also been implemented in tRF research. These tools generally entail adaptations of miRNA target prediction algorithms. tRFtarget predicts mRNA targets of tRFs by combining the results of two prediction tools, RNAhybrid and IntaRNA, which rely on maximum complementary length and free energy between the small RNA and mRNA transcript (119). While these tools provide unbiased scanning of potential mRNA targets, tRFs may exert their effect solely at the proteomic level. The ATtRACT database compiles information on 370 RNA-binding proteins (RBPs) and their 1583 experimentally validated RNA-binding motifs (108). Hence, this tool may help predict potential complexes of tRFs with these well studied RBPs. In the long term, a library of target RNAs and proteins will be constructed for each tRF by compiling RNA-immunoprecipitation data across cells and tissues.

## Roles of piRNAs, snoRNAs and tRFs in the Pathogenesis of Diabetes and Metabolic Disorders

### piRNAs and Metabolism-Related Functions

**Figure 5:**
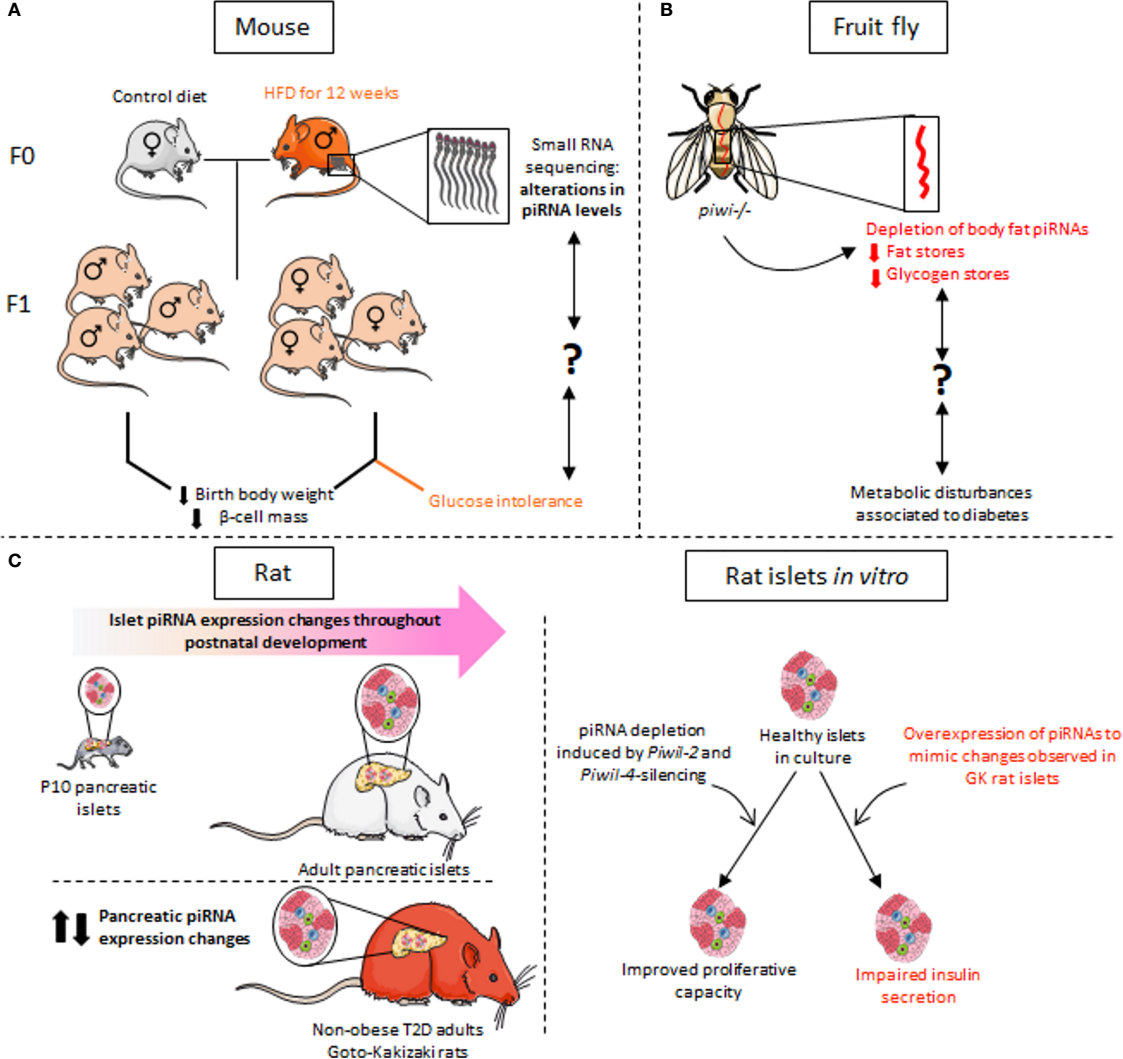
piRNA dysregulation in the context of metabolic disorders. **(A)** RNA-sequencing of sperm from progenitor males fed for 12 weeks on a high-fat diet (HFD) revealed alterations in the expression of many small non-coding RNAs, including piRNAs. F1 animals have reduced birth weight and decreased β-cell mass. Glucose intolerance is observed exclusively in F1 females. **(B)** In a *piwi-mutant Drosophila* model, depletion of piRNAs in the fat body, which has functions equivalent to mammalian liver, results in defects in glucose and lipid metabolism as observed in metabolic disorders such as diabetes. The link between the decline in piRNA expression and the observed metabolic phenotype remains however to be established. **(C)** The expression profile of piRNAs in rat islet cells changes during postnatal development and β-cell maturation. Dysregulation of piRNA levels is also observed in the islets of non-obese diabetic Goto-Kakisaki (GK) rats. *In vitro* experiments mimicking the changes in the level of certain piRNAs observed in GK rats result in defective insulin secretion of healthy rat islets in response to glucose. Depletion of piRNAs secondary to silencing of the PIWI-like genes Piwil2 and Piwil4 in adult islets promotes β-cell proliferation, which is normally very low in adulthood.

piRNAs but also miRNAs and tRFs stand out as key mediators in the transgenerational inheritance of paternal epigenetic marks (see the section more specifically on tRFs regulation in the part named “Identified tRFs and diabetes”). The transgenerational transmission of a metabolic phenotype has been studied using a rat model in which male progenitors (F0) were fed a high-fat diet (HFD) for 12 weeks in adulthood (**Figure 5A**) (120). Newborns of F0 fathers fed HFD displayed reduced birth weight and pancreatic β-cell mass. Exclusively female within the F1 and F2 offspring developed glucose intolerance when the adult animals were challenged by HFD contrary to F1 and F2 females born to F0 fathers fed the control diet. Transcriptomic analysis of the sperm of F0 fathers revealed changes in the expression profile of small ncRNAs including more than thousand piRNAs. Among them, the level of three piRNAs was also changed in the sperm of F1 rats (120). Further studies are needed to understand how paternal transmission of small ncRNAs *via* sperm affects metabolic organs and to demonstrate a causal relationship.

Although less present in somatic cells, piRNAs are detected also in various peripheral tissues and in the central nervous system (121–123). In particular, piRNAs have been found in the fat body of adult Drosophila, an organ that bears functional analogy with mammalian liver (**Figure 5B**). The fat body *piwi null* mutant shows a reduction in fat stores with reduced triacylglycerides and glycogen content (124). piRNAs have also been identified in rat liver (125). The metabolic functions that regulate carbohydrate and lipid homeostasis are clearly disrupted in a context of diabetes. Even though there is currently no established correlation between the above-mentioned data and the impact of liver piRNA changes on the diabetic phenotype, it would be very interesting to dig in this direction. So far, there is only one published study pointing to a causal link between the deregulation of piRNAs and associated PIWI proteins and the loss of pancreatic β-cell function (12). The machinery responsible for piRNA synthesis is clearly present in pancreatic islets since two PIWI-like genes, Piwil2 and Piwil4 were identified in rat islets but also in FACS-sorted rat β-cells and their human orthologues PIWIL2 and PIWIL4 were detected in human pancreatic islets (12). Moreover, piRNAs display a dynamic expression during islet postnatal functional maturation (**Figure 5C**). The levels of some piRNAs were also altered in the islets of Goto-Kakizaki T2D rats. Ectopic overexpression of piRNAs up-regulated in the islets of diabetic rats alters the insulin secretory capacity of healthy β-cells. Moreover, global repression of piRNA levels in adult islet cells secondary to the silencing of piwi proteins promotes proliferation of insulin-secreting cells, known for their low replication capacity (12). The capacity of piRNAs to control replication in other cells types has already been established by various independent studies suggesting that autonomous cellular alteration of piRNA levels is associated with various cancers in mammalian models (126). The resulting loss of transposon repression could result in cancerous cell multiplication as well as a loss of hormone and growth factor action among other cellular pathways.

### snoRNAs in Diabetes and Metabolic Functions

**Figure 6:**
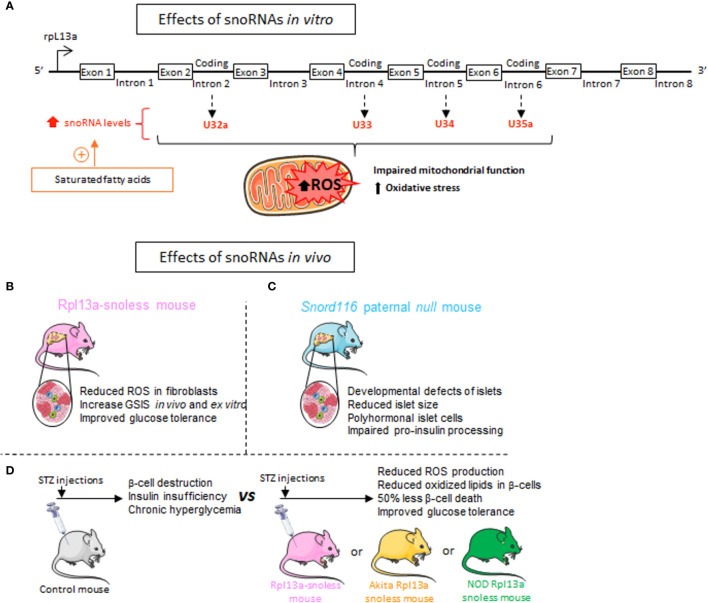
Metabolic effects of snoRNAs. **(A)** Exposure of Chinese hamster ovarian cells and C2C12 Murine Myoblasts to saturated fatty acids including palmitate, results in an increase in the 4 snoRNAs encoded by the introns of Rlp13a gene. These four snoRNAs are known to induce 2’-O-Methylations of target RNAs and mediate palmitate-induced lipotoxic effects on mitochondrial dysfunction and oxidative stress. **(B)** Mice invalidated for the 4 snoRNAs U32a (SNORD32a), U33 (SNORD33), U34 (SNORD34), and U35a (SNORD 35a) named Rpl13a-snoless mice, display reduced Reactive Oxygen Species (ROS) production, increased insulin secretion and improved glucose tolerance. **(C)** *Rpl13a*-snoless mice treated with the β-cell death inducer Streptozotocin (STZ) or bred with *Ins2C96Y* Akita mice or non-obese diabetic (NOD) mice are protected against the development of chronic hyperglycemia, show less oxidative stress and improved glucose tolerance. **(D)** Patients with Prader-Willi syndrome (PWS) who develop severe obesity and metabolic disorders carry a loss of paternally expressed genes on chromosome 15 which encodes for SNORD116. To reproduce the genetic background of PWS patients, a paternal *Snord116* mouse knockout (*Snord116p–/m+*) has been generated. Genetic deletion of SNORD116 leads to defects in pancreatic endocrine development along with metabolic disturbances.

The distribution of snoRNAs appears to be specific within various tissues and sensitive to the environment (43). In response to exposure to pro-inflammatory saturated fatty acids, including palmitate, stearate or myristate, the expression of 4 snoRNAs [box C/D snoRNAs U32a (SNORD32a), U33 (SNORD33), U34 (SNORD34), and U35a (SNORD 35a)] known to introduce 2’-O-Methylations in target RNAs is increased (**Figure 6A**) (127). Disruption of the locus ribosomal protein L13a (rpL13a), which encodes for three of these box C/D small nucleolar RNAs (snoRNAs 32a, 33 and 35) from intronic regions, confers resistance to lipotoxicity to an ovarian cell line. The loss of these snoRNAs, rather than the RPL13A protein, is responsible for resistance to oxidative stress and mitochondrial dysfunction secondary to exposure to high lipid concentrations (**Figure 6A**) (127). The effect of these snoRNAs in propagating oxidative stress in the liver of mice treated with the inflammatory agent Lipopolysaccharide (LPS) has also been demonstrated *in vivo* (127). Additional findings are consistent with these observations and link this phenotype to a physiopathological context. A mouse model (Rpl13a-snoless mice) in which four snoRNAs-hosting introns (encoding for SNORD32a, -33, -34 and -35) have been specifically deleted without disturbing the expression of the ribosomal protein Rpl13a has been generated (128). The absence of these four snoRNAs leads to a drop in the level of reactive oxygen species (ROS), resulting in an increase in insulin secretion in response to glucose from pancreatic islets and an improvement in glucose tolerance (**Figure 6B**) (128). Since ROS have been shown to be involved in diabetes pathogenesis in humans and various animal models, the authors evaluated the resistance of the mice to diabetes development following the administration of low doses of streptozotocin (STZ), an agent that causes oxidative stress and β-cells death. Upon STZ treatment, Rpl13a-snoless mice displayed improved glucose tolerance compared to control mice. Histological analysis of the pancreas revealed that β-cells of Rpl13a-snoless mice are protected from STZ-induced accumulation of oxidized lipids that are toxic to insulin-secreting cells and show 50% less β-cell death compared to control animals. Similar results were obtained from two other mouse models showing oxidative damage in β-cells leading to apoptosis and development of diabetes, including Akita mice carrying the Ins2C96Y allele and non-obese diabetic mice (NOD). This study provides evidence that Rpl13a snoRNAs contribute to the hyperglycemia observed in three mouse diabetes models presenting an inflammatory context associated with β-cell demise (**Figure 6C**) (128).

Being mainly known to guide post-transcriptional modifications of ribosomal and small nuclear RNAs, snoRNAs resonate as potentially vital factors in the drastic reshaping of the transcriptional and proteomic landscape associated with metabolic diseases (7, 129). Various independent studies revealed a contribution of snoRNAs to the control of food intake and to body weight regulation in patients suffering from the neurodevelopmental disorder called Prader-Willi syndrome (PWS). PWS is a multifactorial pathology leading to morbid obesity and metabolic syndrome (130). The units coding for the orphan snoRNAs SNORD115 and SNORD116 reside within an intronic region of chromosome 15 whose deletion results in the development of PWS (131, 132). Concomitant overexpression of these two snoRNAs in HEK293T cells induces changes in the level of more than twenty transcripts corresponding to genes whose expression is also disrupted in the brains of subjects with PWS (133). The mechanisms underlying this effect are not yet known and it remains to be determined whether SNORD115 and SNORD116 directly affect the stability of the identified mRNAs or act *via* an intermediate trans-acting factor. *In vivo*, mice invalidated for SNORD116 display major metabolic alterations, including a defect in the development of the endocrine pancreas (134) and in proinsulin processing (**Figure 6D**) (135). The islets of these mice are smaller with an increase in the number of δ-cells secreting the insulin antagonist hormone somatostatin and a decrease in glucagon-secreting α-cells. Moreover, markers of β-cell differentiation and function such as *Ins1*, *Ins2*, *Pdx1*, *Nkx6-1*, and *Pax6* are reduced (134).

Alongside, both PWS and diabetic patients have alterations in circadian rhythms defined as a misalignment between the endogenous clock system and behavioral circadian cycles (for example, sleep-wake and fasting-feeding cycles) (136, 137). Several studies have investigated the rhythmic expression of snoRNAs in various organisms and tissues (79, 138, 139). In mice, genetic deletion within the SNORD116/Snord116 region affects sleep physiology and suggests a role for the small nucleolar ribonucleic acid-116 (SNORD116) in sleep alterations involving the rapid eye movement (REM) phase which is perturbed in most patients with PWS (140, 141). In light of these data, snoRNAs could be key regulators of the molecular pathways contributing to rhythmic transcriptome and proteome defects associated with diabetes. Taken together, these findings implicate snoRNAs in the development of metabolic disorders and pave the way for the discovery of new factors causing chronic multifactorial endocrine diseases.

Not only the expression of snoRNAs is dynamic in response to various exogenous stimuli, but also their location is sensitive to environmental factors. The release of snoRNAs into the bloodstream by macrophages *via* extracellular vesicles results in an inflammatory context in mouse models and human subjects (84). The authors of this study showed using parabiosis experiments that snoRNAs released into the circulation are capable of inducing 2’-O-methylations of RNAs in various peripheral tissues including enterocytes. How tissues are specifically targeted by vesicles carrying snoRNAs and whether these small RNAs significantly contribute to the immune response induced in recipient cells remains to date unknown.

### Identified tRFs and Diabetes

**Figure 7:**
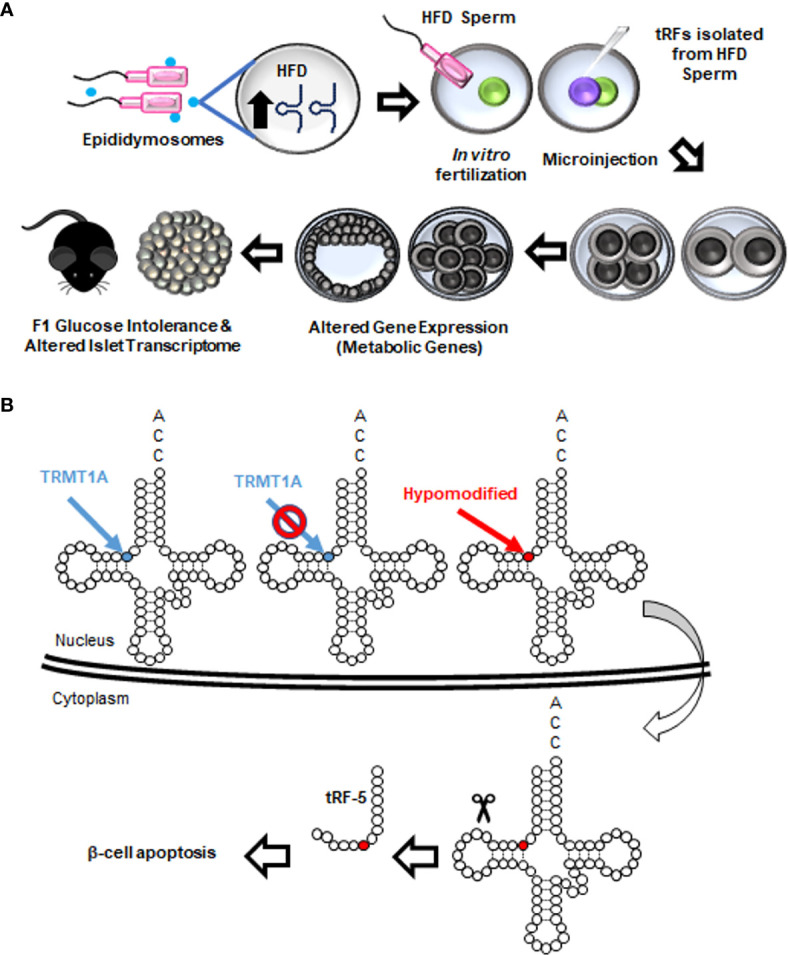
Roles of tRFs in metabolic disorders and β-cell dysfunction. **(A)** Paternal high fat diet (HFD) increases the abundance of tiRNA-5s that are packaged inside epididymis-derived exosomes (epididymosomes). Epididymosomes fuse with the sperm and transfer these fragments. When normal zygotes are fertilized with HFD sperm or microinjected with tRFs isolated from HFD sperm, expression of metabolic genes in eight-cell and blastocyst-stage embryos is drastically altered. The resulting offspring exhibit abnormal metabolism at adulthood, including glucose intolerance and alterations in pancreatic islet transcriptome. A similar metabolic function of tRFs is observed upon a paternal low protein diet. **(B)** TRMT10A deficiency is associated with hypomethylation of guanosine residues at position 9 of certain tRNAs. Hypomethylated tRNAs are transported to the cytoplasm and fragmented. tRF-5s generated from such fragmentations lead to β-cell apoptosis.

Intergenerational inheritance of metabolic disorders and β-cell dysfunction are established (142–144). In recent years, paternal metabolic challenges were shown to be correlated with alterations in sperm tRFs in humans, mice, and rats (111, 120, 145, 146). Importantly, isolation of sperm tRFs from mice fed a high fat diet and injection of these tRFs into healthy zygotes was sufficient to dysregulate islet transcriptome and trigger an obesogenic phenotype in F1 mice (**Figure 7A**) (145). In line with this observation, another study illustrated that maternal high fat diet induces an obesogenic phenotype in F1, and injection of tRFs isolated from F1 into healthy zygotes reproduced the phenotype observed in F1 (147). Altogether, these studies underline the contribution of tRFs to intergenerational inheritance of diet-induced metabolic disorders. Mechanistically, high fat diet altered the methylation status and stability of tRFs which in turn repressed a subset of metabolic genes at an embryonic stage, in a miRNA-like fashion (145). Accordingly, tiRNA-5^GluCTC^, a diet-regulated sperm tRF, was enriched upon Ago2 immunoprecipitation (148).

While the impact of sperm tRFs on the offspring’s metabolism is rapidly unraveling, the tRF landscape and function in β-cells remain largely elusive. The only study on tRF function in β-cells was recently published in the context of monogenic mutations in *TRMT10A* gene encoding for a tRNA methyltransferase (149). This mutation causes young onset diabetes and microcephaly (150). In β-cells, TRMT10A deficiency was shown to cause oxidative stress and trigger apoptosis. However, the mechanism underlying the β-cell apoptosis was unknown. In the study cited above (149), glutamine tRNAs were shown to be hypomethylated in the absence of TRMT10A, leading to increased tRNA fragmentation and accumulation of tRF-5s. Interestingly, transfecting the EndoC-H1 human β-cell line with glutamine tRF-5 mimics increased β-cell apoptosis. Conversely, inhibition of the fragment in *TRMT10A* deficient β-cells decreased apoptosis, suggesting that β-cell death observed in *TRMT10A* mutations may be attributed to this tRF (**Figure 7B**). While the exact mechanism of tRF-induced β-cell apoptosis is unknown, it can be speculated that the tRF is interacting with Cytochrome c to regulate this process, as reported in other models (118).

Since β-cell death is a key event in T1D development, future studies in T1D models, such as NOD mice, may help elucidate the involvement of tRFs in this form of the disease.

## Conclusion and Future Perspectives

The last few years have witnessed tremendous advancements in the understanding of the physiological role of different classes of ncRNAs and of their involvement in the etiology of multifactorial pathologies such as diabetes. Among them, the newly identified piRNAs, snoRNAs and tRFs are emerging as important regulators of signaling pathways activated or affected during the development of diabetes, thus placing them as potential therapeutic targets for the treatment of this metabolic disease. The capacity of ncRNAs to control the stability of genomic elements or transcripts but also to modulate several downstream pathways can drastically impact the molecular and functional landscape of the cells, justifying efforts to develop drugs specifically targeting these molecules. Pre-clinical studies involving the use of synthetic miRNA-based therapeutic molecules have already shown remarkable and very promising results in term of ability to reach and penetrate cells (hepatocytes, tumor cells, etc.) and to accomplish relevant regulatory activities (151). Early-phase human clinical trials that target miRNAs raise a lot of hope on the feasibility of applying similar strategies to modulate also the level or the function of other classes of small ncRNAs such as piRNAs, snoRNAs and tRFs in diabetes therapy. The strategy employed is to modulate ncRNA levels and/or activity in either insulin-secreting β-cells or in other cells implicated in the pathogenesis of diabetes (other islet cell types, insulin target tissues, central nervous system) (151). For over-active ncRNAs, the approach consists in using antisense oligonucleotides or CRISPR/Cas9 in order to attenuate or block the effects of the selected ncRNA. For palliating the absence or the inhibitory activity of ncRNAs, a replacement therapy can be envisaged *via* synthetic oligonucleotides mimicking the ncRNA sequence. The injection of “mimic” or “inhibitor” molecules to the targeted tissue can be mediated by protective delivery methods such as lipid-based nanoparticles, exosomes, or polymer-based delivery systems (152–155). Some ncRNAs are specifically expressed or selectively up or down-regulated in a particular cell type depending on the pathophysiological context. Since each ncRNA can play potentially different roles according to the host cell, the real challenge will be to successfully target a specific cell type and thus minimize the side effects that could be caused by ubiquitous modulation of the ncRNA.

We are only beginning to appreciate the role of piRNAs, snoRNAs and tRFs in pancreatic β-cells and in metabolic tissues and their possible involvement in different forms of diabetes. A better understanding of the mode of action and of the contribution of these small ncRNAs to the etiology of diabetes will help identifying potential targets for the development of new pharmaceutical principles to prevent or treat this metabolic disorder. The design of drugs to modulate the level or the activity of piRNAs, snoRNAs or tRFs will certainly take advantage of the knowledge gathered from the growing interest for siRNA- or miRNA-based therapeutic approaches, a field of intense investigation. Thus, there is hope that if relevant piRNAs, snoRNAs or tRFs targets will be identified, the development of therapeutic approaches to modulate the activities of these small ncRNAs will be greatly accelerated.

## Author Contributions

CJ, MB, and RR searched the literature and wrote the article. All authors contributed to the article and approved the submitted version.

## Funding

The authors are supported by a grant from the Swiss National Science Foundation (310030_188447).

## Conflict of Interest

The authors declare that the research was conducted in the absence of any commercial or financial relationships that could be construed as a potential conflict of interest.
